# Induction of P-glycoprotein overexpression in brain endothelial cells as a model to study blood-brain barrier efflux transport

**DOI:** 10.3389/fddev.2024.1433453

**Published:** 2024-07-05

**Authors:** Sarah F. Hathcock, Hallie E. Knight, Emma G. Tong, Alexandra E. Meyer, Henry D. Mauser, Nadine Vollmuth, Brandon J. Kim

**Affiliations:** ^1^ Department of Biological Sciences, University of Alabama, Tuscaloosa, AL, United States; ^2^ Department of Microbiology, Heersink School of Medicine, University of Alabama at Birmingham, Birmingham, AL, United States; ^3^ Center for Convergent Biosciences and Medicine, University of Alabama, Tuscaloosa, AL, United States; ^4^ Alabama Life Research Institute, University of Alabama, Tuscaloosa, AL, United States

**Keywords:** blood-brain barrier, P-glycoprotein, efflux transport, drug screening, brain endothelial cells, puromycin, drug delivery

## Abstract

The blood-brain barrier (BBB) is comprised of specialized brain endothelial cells (BECs) that contribute to maintaining central nervous system (CNS) homeostasis. BECs possess properties such as an array of multi-drug efflux transporters that eject various drugs and toxins, preventing their entry into the CNS. Together, it is estimated that these efflux transporters can eject up to 98% of known xenobiotic compounds. P-glycoprotein (P-gp) is a promiscuous efflux transporter at the BBB and can efflux up to 90 various substrates, representing a major hurdle in CNS drug delivery for therapeutic interventions. This necessitates the study of P-gp to discover drugs that are non-substrates of P-gp as well as to identify novel P-gp inhibitors. Here we report the generation of P-gp overexpressing BECs under the endogenous promoter control that could be used in the screening of P-gp substrates. These cells could provide utility in the design of drugs or identification of novel inhibitors.

## Introduction

The blood-brain barrier (BBB) is comprised of highly specialized brain endothelial cells (BECs) that maintain central nervous system (CNS) homeostasis by tightly regulating the passage of molecules from the circulation (Engelhardt and Sorokin, 2009; Abbott et al., 2010; Redzic, 2011). BECs are unique compared to peripheral endothelial cells as they possess complex tight junctions, exhibit low rates of endocytosis and transcytosis, lack fenestrations in the capillaries, and express multi-drug efflux transporters (Engelhardt and Sorokin, 2009; Abbott et al., 2010; Redzic, 2011). P-glycoprotein (P-gp/MDR1) is a major efflux transporter found at the BBB and other tissues such as the gut and the blood-placental barrier (Mahar et al., 2002; Bauer et al., 2005; Perriere et al., 2005; van Assema et al., 2012; Davis et al., 2014). Generally, P-gp excludes small lipophilic molecules and some large molecules and proteins such as the amyloid- β peptide implicated in Alzheimer’s disease (Mahar et al., 2002; Bauer et al., 2005; van Assema et al., 2012; Amin, 2013; Davis et al., 2014). Typically, P-gp is apically localized on polarized epithelial barriers and the BBB to facilitate efflux of substrates, excluding them from the tissue (Cordon-Cardo et al., 1989; Thiebaut et al., 1989; Schinkel, 1999). P-gp remains a major hurdle for the delivery of potentially therapeutic drugs to the CNS as it can restrict their passage and accumulation (Azad et al., 2015; Chatterjee et al., 2023; Cox et al., 2023). Therefore, the study of P-gp remains of high interest. Other *in vitro* models provide utility in examining P-gp including cell lines such as the MDCK-MDR1 overexpression line and stem-cell derived brain-like endothelial cells (Braun et al., 2000; Liu and Zeng, 2008; Lippmann et al., 2012; Cecchelli et al., 2014). However the MDCK-MDR1 cell line relies on a retroviral promoter to drive human P-gp expression, and the stem-cell derived models may have limited transporter expression depending on which parent stem-cell line is used (Pastan et al., 1988; Liu and Zeng, 2008). Previous work has demonstrated that application of cytotoxic P-gp substrates increases the purity of rodent primary BECs and induces P-gp expression in primary rat brain endothelial cells (Demeuse et al., 2004; Stebbins et al., 2016; Espinal et al., 2022). Here we sought to generate an endogenous P-gp overexpression cell line in the well characterized brain endothelial cell model, hCMEC/D3 (immortalized cerebral microvascular endothelial cells) (Weksler et al., 2005). Using the cytotoxic P-gp substrate puromycin, we evolved the hCMEC/D3 cell line to select for cells that highly express P-gp (hCMEC/D3-MDR1). hCMEC/D3-MDR1 cells express P-gp at high levels under the endogenous promoter control of brain endothelial cells and display a large dynamic range for P-gp activity assays. These hCMEC/D3-MDR1s provide a useful tool for screening large molecular libraries for novel P-gp inhibitors that could enhance drug delivery to the CNS.

## Methods

### Maintenance of hCMEC/D3 and generation of P-gp overexpressing hCMEC/D3-MDR1 cells

hCMEC/D3 cells were maintained in EndoGRO MV (Millipore) media as previously described (Weksler et al., 2005; Espinal et al., 2022). Briefly, cells were passaged onto new flasks coated with 1% rat tail collagen (RTC) at 80% confluency. Generation of P-gp overexpressing hCMEC/D3 cells (hCMEC/D3-MDR1) was initiated by passing naïve hCMEC/D3s into media containing 0.5 μg/mL puromycin (AdipoGen cat# AG-CN2-0078-M025). Surviving cells were passed for five passages in 0.5 μg/mL puromycin before switching the media to 1 μg/mL puromycin. hCMEC/D3-MDR1s were stocked, frozen, and maintained in 1 μg/mL puromycin. All cells were incubated at 37°C + 5% CO_2_ in EndoGRO MV media with or without puromycin as described above, and media was changed on cells to EndoGRO MV without puromycin 1 day before any downstream applications.

### Transendothelial electrical resistance

Transendothelial electrical resistance (TEER) was measured using an EVOM II (World Precision Instruments) and the chopstick electrodes associated. hCMEC/D3 or hCMEC/D3-MDR1s were seeded onto Corning Transwells (3460) coated in 1% RTC and grown for 2 days at 37°C + 5% CO_2_ in EndoGRO MV media with or without puromycin as described above. Measurements were taken each day following seeding for 5 days.

### Quantitative PCR

hCMEC/D3s or hCMEC/D3-MDR1s were seeded onto 1% RTC coated 24-well plates in triplicate and grown for 4–5 days at 37°C + 5% CO_2_ in EndoGRO MV media with or without puromycin as described above. RNA was collected and purified using the Machery-Nagel NucleoSpin RNA kit (Fisher cat# 740955.250) and concentrations were measured using a Nanodrop 2000. Matching amounts of purified RNA were converted to cDNA using the qScript kit (Quantabio cat# 95047-500) and diluted 1:10 in nuclease-free water to be used for qPCR. SYBR green (Thermo Fisher cat# A25743) qPCR was performed on cDNA and measured using a QuantStudio3 (Thermo Fisher). Delta-delta CT calculations were made using 18*S* as a housekeeping gene. Data are expressed as fold change compared to naïve cells.

### Flow cytometry

hCMEC/D3s or hCMEC/D3-MDR1s were seeded onto 1% RTC coated 25 cm^2^ flasks and grown for 4–5 days at 37°C + 5% CO_2_ in EndoGRO MV media with or without puromycin as described above. Cells were washed 3 times with PBS and removed from the flask by applying 1 mL Accutase™ (Stemcell Technologies cat# 07920) and incubating at 37°C + 5% CO_2_ for 10 min. Cells were collected with 4 mL of fresh EndoGRO MV media and pelleted at 500 × g for 10 min. Cells were fixed with 1% formaldehyde in PBS for 15 min at RT. Cells were then pelleted and washed twice with wash buffer (5% bovine serum albumin (BSA) + 0.1% Triton-X in PBS) to block and permeabilize for staining of total P-gp. Cells were resuspended in 1 mL wash buffer and cell density was estimated using a Countess 3 Automated Cell Counter. Samples were stained in wash buffer with anti-P-gp (Thermo Fisher cat# MA5-13854 [F4]) or an IgG1 isotype (Bio X Cell cat# BE0083) at .05 μg/million cells, and gently agitated overnight at 4°C. The following day, cells were washed twice in wash buffer and stained with a fluorescent secondary antibody (anti-mouse Alexa fluor 488, Thermo cat# A11001) at 1:5000 in wash buffer for 1 h at room temperature. Cells were then washed in wash buffer and resuspended in PBS. Data was collected on an Attune NxT Flow Cytometer, and histograms and dot plots were generated using the Attune NxT Software.

### Western blot

hCMEC/D3s and hCMEC/D3-MDR1s were seeded onto 1% RTC coated 24-well plates and grown for 4–5 days at 37°C + 5% CO_2_. Samples were lysed in RIPA buffer containing protease inhibitor cocktail and protein abundance was measured by bicinchoninic acid (BCA) assay (Thermo Fisher cat# 23227). Equal amounts of protein were then loaded on to 4%–12% Tris-glycine SDS-PAGE gels followed by transfer to a nitrocellulose membrane. Membranes were then stained by Ponceau S stain and imaged prior to blocking in 5% milk in Tris-buffered saline +0.1% Tween (TBST) for 1 h at room temperature. Primary antibodies diluted in 5% milk in TBST were then applied and allowed to incubate with the membrane overnight at 4°C. The following day, membranes were washed three times in TBST for 5 minutes each, followed by incubation with appropriate secondary antibodies (anti-mouse HRP Jackson ImmunoResearch cat# 115-035-003) (1:1000) for at least 1 h at room temperature. Following incubation, membranes were washed three times in TBST for 5 minutes each and imaged using enhanced chemiluminescence substrate (Thermo Fisher cat# 34577) and an iBright imager. Primary antibodies used: anti-VE-Cadherin (Santa Cruz cat# sc-52752 [BV9]) (1:1000), anti-GLUT1 (Thermo Fisher cat# MA5-11315 [SPM-498]) (1:1000), anti-P-glycoprotein (Thermo Fisher cat# PA5-28801]) (1:1000), anti-PECAM (CD31) (Thermo Fisher cat#BMS137 [Gi18]) (1:1000).

### Substrate accumulation assays

Substrate accumulation assays were performed as described previously (Stebbins et al., 2016; Kim et al., 2019). For P-gp substrate accumulation, Rhodamine 123 (R123) (Sigma-Aldrich 83702) was utilized to measure relative P-gp function. Briefly, cells were washed in Hank’s Balanced Salt Solution (with Calcium and Magnesium) (Thermo Fisher 14065056) and incubated with or without the inhibitors cyclosporine A (CsA) (Sigma Aldrich C1832) or Valspodar (PSC833) (R&D systems 40-421) at 10 μM for 1 h at 37°C + 5% CO_2_. Following pre-incubation, cells were then treated with 10 μM R123 with or without inhibitors and incubated for 2 h at 37°C + 5% CO_2_. Following incubation, cells were washed twice in ice-cold PBS and lysed in RIPA buffer. Samples were then measured using a SpectraMax iD3 plate reader (Molecular Devices), and BCA assays (Thermo Fisher cat# 23227) were then conducted on samples from each well. Fluorescence accumulation measurements were normalized to protein amounts.

### Microscopy

hCMEC/D3s and hCMEC/D3-MDR1s were seeded onto 1% RTC coated 24-well plates and grown for 4–5 days at 37°C + 5% CO_2_. Cells were then imaged on a Nikon Ti2 microscope using a 20X objective and images were processed using ImageJ software (scale bar represents 50 µm).

### Statistics

GraphPad Prism version 10.2.1 was used for all statistics calculations. For pair-wise comparisons, Student’s t tests were performed. Statistical significance was accepted if *p* < 0.05.

## Results

### Generation of hCMEC/D3-MDR1 from hCMEC/D3 immortalized brain endothelial cells

**FIGURE 1:**
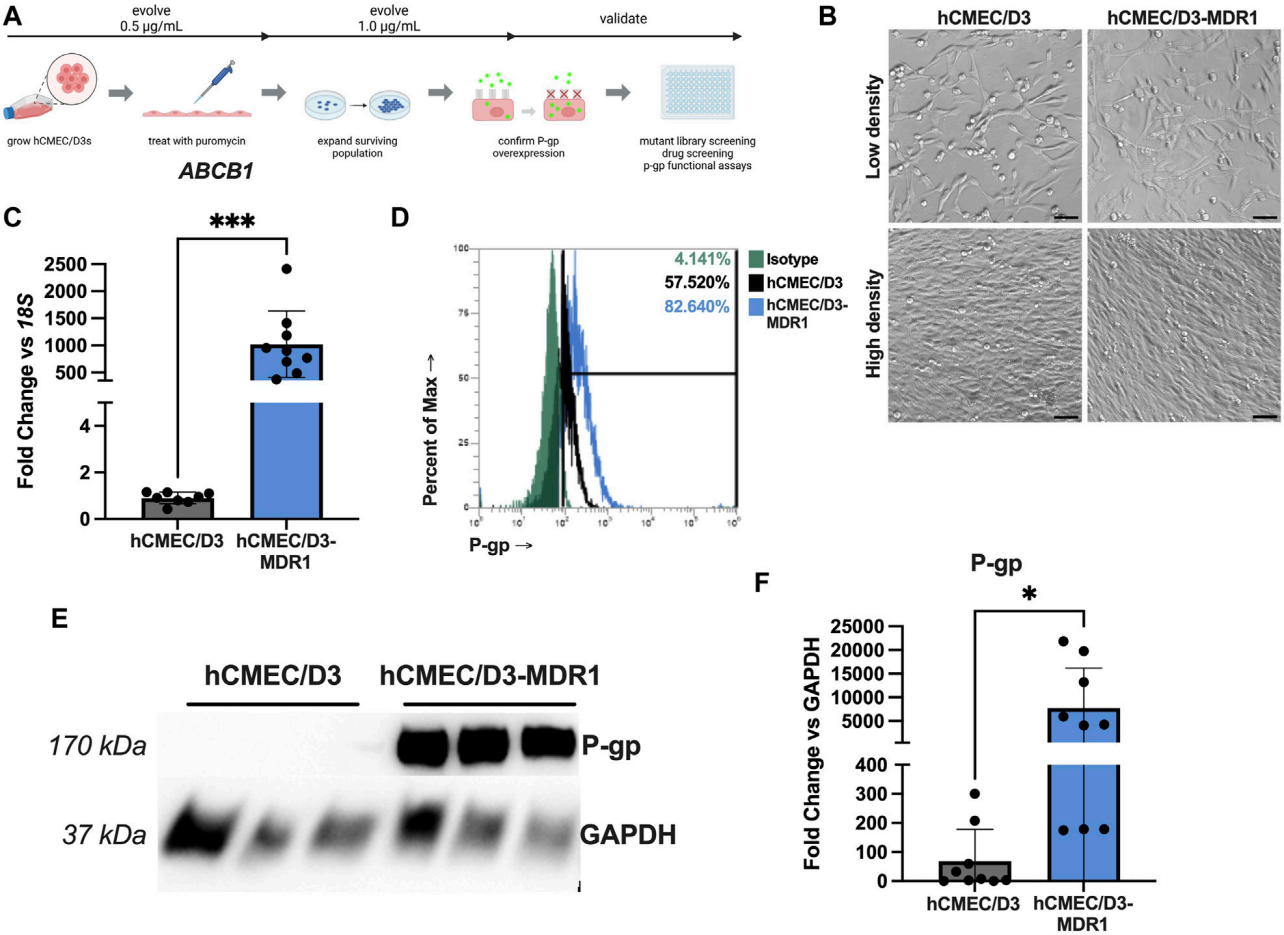
*hCMEC/D3-MDR1 can be maintained in puromycin media and overexpress P-gp* **(A)** Schematic of selection of hCMEC/D3-MDR1 and downstream applications (Created with Biorender.com), **(B)** Differential interference contrast microscopy of hCMEC/D3s *versus* hCMEC/D3-MDR1s at low and high densities reveal similar morphology between cell types, **(C)** qPCR of *ABCB1* in hCMEC/D3s (gray) *versus* hCMEC/D3-MDR1s (blue) reveal upregulation of P-gp transcripts in hCMEC/D3-MDR1, **(D)** Flow cytometry reveal increased total P-gp in hCMEC/D3-MDR1 (blue = P-gp; green = isotype) *versus* hCMEC/D3 (black = P-gp) populations, **(E)** Western blot of P-gp in hCMEC/D3s *versus* hCMEC/D3-MDR1s reveal increased P-gp abundance in hCMEC/D3-MDR1 quantified in **(F).** Scale bars represent 50 µm. Student’s t*-*test was performed; *p < 0.05, ***p < 0.001. Experiments were performed on three distinct passages and in technical triplicate (*n* = 8 or 9) **(C–F)**; representative images are shown.

hCMEC/D3 cells are typically maintained in EndoGRO MV media. To generate hCMEC/D3-MDR1, we chose to select for highly expressing P-gp cells by subjecting cells to the cytotoxic P-gp substrate puromycin. This strategy has been utilized previously to selectively purify BECs from other cell types in the brain as BECs expressing P-gp will efflux the toxin and survive (Demeuse et al., 2004; Perriere et al., 2005; Calabria et al., 2006). To select for highly expressing P-gp cells, hCMEC/D3 cells were passaged into flasks containing 0.5 μg/mL puromycin and allowed to expand. After at least five passages in 0.5 μg/mL, these were then passaged into 1 μg/mL puromycin (Figure 1A). hCMEC/D3 and hCMEC/D3-MDR1 possess similar morphology at low and high densities (Figure 1B). Expression of endogenous P-gp was measured by qPCR, which demonstrated that hCMEC/D3-MDR1 significantly upregulate *ABCB1* (P-gp) compared to naïve hCMEC/D3s (Figure 1C). Examination of P-gp protein by flow cytometry (Figure 1D, Supplementary Figure S1) and immunoblot show increased levels of P-gp protein abundance in hCMEC/D3-MDR1 when compared to naïve hCMEC/D3s (Figures 1E, F). Levels of TEER remained similar between naïve hCMEC/D3 and hCMEC/D3-MDR1s once confluence was reached (Supplementary Figure S2A). Other markers of endothelial identity were slightly decreased in the hCMEC/D3-MDR1s *versus* the naïve hCMEC/D3s, indicating a partial loss of endothelial identity (Supplementary Figures S2B, C). Taken together these data demonstrate that the hCMEC/D3-MDR1s can be maintained in puromycin and overexpress P-gp without any exogenous genetic manipulation.

### hCMEC/D3-MDR1 overexpress functional P-gp measured by substrate accumulation assays

**FIGURE 2:**
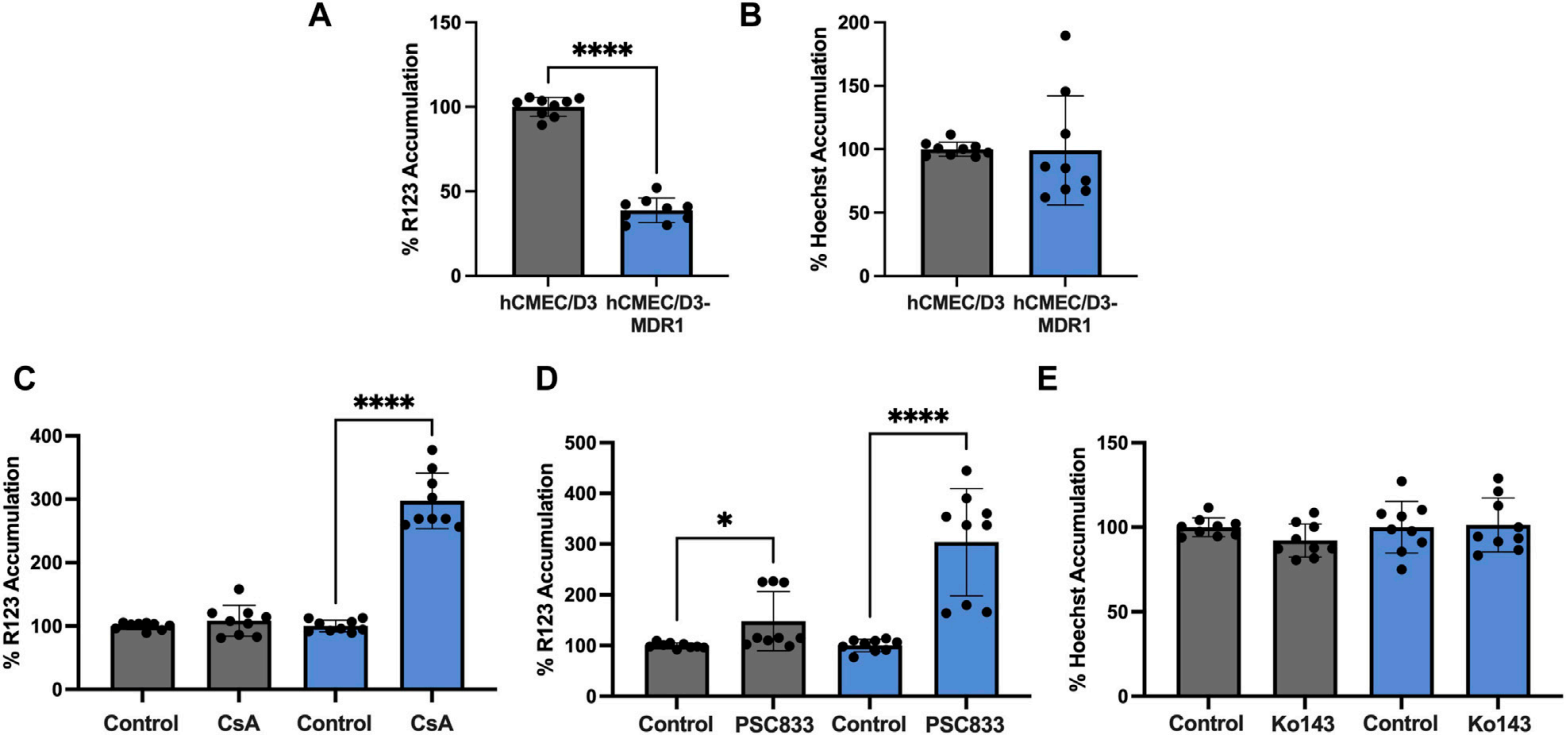
*hCMEC/D3-MDR1 display increased P-gp specific function* **(A)** Substrate accumulation of 10 µM R123 is significantly diminished in hCMEC/D3-MDR1 (blue) *versus* hCMEC/D3 (gray) indicating increased total P-gp mediated efflux, **(B)** Substrate accumulation of Hoechst does not differ between hCMEC/D3-MDR1s and hCMEC/D3s, indicating no change in BCRP mediated exclusion, substrate accumulation of R123 in the presence of P-gp inhibitors 10 µM cyclosporine A **(C)** or 10 µM PSC833 **(D)** indicate an increased dynamic range in P-gp inhibition in hCMEC/D3-MDR1s *versus* hCMEC/D3s, **(E)** Substrate accumulation of 10 µM Hoechst in the presence of BCRP inhibitor 1 µM Ko143 indicate no difference in the dynamic range of BCRP inhibition of hCMEC/D3-MDR1s *versus* hCMEC/D3s. All data are expressed as percent accumulation of control. Student’s t*-*test was performed; *p < 0.05, ****p < 0.0001. Experiments were performed on three distinct passages and in technical triplicate (*n* = 9).

We have demonstrated that hCMEC/D3-MDR1 overexpress P-gp (Figure 1). Next, to assess if this overexpression leads to a functional exclusion of substrates, we applied a well characterized substrate accumulation assay. Typically, P-gp excludes Rhodamine 123 (R123) from cells; however, if P-gp is inhibited with pharmacological inhibitors such as cyclosporine A (CsA) or the second-generation P-gp inhibitor Valspodar (PSC833), R123 is able to accumulate inside cells. To determine if another transporter, Breast Cancer Resistance Protein (BCRP), was functionally changed during the generation of hCMEC/D3-MDR1, we employed a similar substrate accumulation assay using the BCRP substrate Hoechst and inhibitor Ko143. Overall accumulation of R123 in hCMEC/D3 *versus* hCMEC/D3-MDR1 reveal that hCMEC/D3-MDR1 exclude R123 significantly more efficiently than hCMEC/D3 (Figure 2A). We also found no change in Hoechst exclusion between cell types (Figure 2B). We found that hCMEC/D3s minimally exclude R123 even in the absence of CsA or PSC833 (Figures 2C, D). However, hCMEC/D3-MDR1s significantly exclude R123 in the absence of an inhibitor and accumulate R123 in the presence of both inhibitors, suggesting that functional P-gp is highly expressed in hCMEC/D3-MDR1 (Figures 2C, D). Finally, we found that hCMEC/D3s and hCMEC/D3-MDR1s exclude Hoechst at similar rates, even in the presence of Ko143 (Figure 2E). These data suggest that hCMEC/D3-MDR1 overexpress functional P-gp and that the generation of hCMEC/D3-MDR1 did not impact BCRP expression or function. Therefore, we conclude that the hCMEC/D3-MDR1 will have particular utility in the screening for P-gp specific inhibitors or substrates.

## Discussion

Drug delivery to the CNS remains a major hurdle to overcome for the treatment of neurological disorders (Azad et al., 2015; Bhunia et al., 2023; Chatterjee et al., 2023; Cox et al., 2023; Ronaldson and Davis, 2024). The BBB restricts the majority of small molecules from entering the CNS due in part to the expression of functional efflux transporters (Azad et al., 2015; Bhunia et al., 2023; Chatterjee et al., 2023; Cox et al., 2023; Ronaldson and Davis, 2024). P-gp is a major efflux transporter present in BECs that contributes to the efflux of many drugs and toxins, preventing their entry to the CNS (Azad et al., 2015; Chatterjee et al., 2023; Cox et al., 2023). Here we report on a cell line that could be used to screen drugs and potential P-gp inhibitors (Figure 1A) that is of BEC origin and controls P-gp under the endogenous promoter.

Since the cytotoxic puromycin is a substrate for P-gp and has been utilized to purify BECs from primary tissue, we selectively expanded hCMEC/D3s that highly express P-gp and protect themselves from puromycin’s toxic impacts (Demeuse et al., 2004; Perriere et al., 2005; Calabria et al., 2006). These hCMEC/D3-MDR1s highly express functional P-gp and retain most characteristics of the parent hCMEC/D3 cell line (Figures 1, 2). Other P-gp overexpressing cells such as the MDCK-MDR1 cell line use exogenous promoters and genes to achieve this goal (Braun et al., 2000; Liu and Zeng, 2008; Lippmann et al., 2012; Cecchelli et al., 2014). These models are highly useful as they generate polarized monolayers, and when grown on transwells, can indicate whether a particular drug is a P-gp substrate as measured by drug concentrations between the apical and basolateral sides. Additionally, as these cells provide a polarized monolayer, they can mimic the properties of many cellular barriers, primarily epithelial. hCMEC/D3 cells are of endothelial origin and since they do not possess extremely tight barrier properties they may not be the most useful for drug delivery studies (Weksler et al., 2005; Helms et al., 2016). While the hCMEC/D3-MDR1s also do not possess tight barrier properties, they could still prove useful in the screening of potential P-gp inhibitors on endothelial cells. Furthermore, although endothelial properties are slightly altered in hCMEC/D3-MDR1 (Supplementary Figure S2), functional efflux transport still remains (Figure 2), preserving the utility of the model. hCMEC/D3-MDR1s control P-gp expression under the endogenous promoter and are of endothelial origin. Other endothelial models that demonstrate P-gp function include induced pluripotent stem-cell (iPSC) derived brain-like endothelial cell models (iBECs) (Pastan et al., 1988; Liu and Zeng, 2008). However, there are some iPSC lines that, even after differentiation, do not seem to possess robust transporter function (Ozgur et al., 2023; Nielsen et al., 2024). iBECs that do possess efflux transporter function demonstrate a roughly 50%–100% increase in R123 substrate accumulation when subjected to CsA or PSC833 (Lippmann et al., 2012; Lippmann et al., 2014; Stebbins et al., 2016; Hollmann et al., 2017; Kim et al., 2019; Neal et al., 2019). In contrast, the hCMEC/D3-MDR1s exhibit a dynamic range in the same assay of nearly 300% (Figure 2), potentially increasing the signal-to-noise ratio required to screen for novel P-gp inhibitors on endothelial cells. Overall, we report that the hCMEC/D3-MDR1 cell line robustly expresses functional P-gp and could be used for the screening of drug libraries or microbial mutant libraries for novel P-gp inhibitors in a native endothelial cell.

## Data Availability

The original contributions presented in the study are included in the article/Supplementary Material, further inquiries can be directed to the corresponding author.

## Supplementary material

SUPPLEMENTARY FIGURE S1
**(A)** Representative flow cytometry with gating for unstained population reveals a larger shift into the gate for P-gp positive hCMEC/D3-MDR1s versus hCMEC/D3s with total mean gated percentages represented in **(B)** Student's t test was performed; * *p* < 0.05. Experiments were performed on three distinct passages and in technical triplicate (*n* = 9).

SUPPLEMENTARY FIGURE S2
*hCMEC/D3-MDR1 display diminished endothelial identity* **(A)** TEER remains at similar levels between hCMEC/D3s (gray) and hCMEC/D3-MDR1 (blue) at confluence, **(B)** Western blots of VE-Cadherin, CD31, and GLUT1 reveal trending diminished abundance of CD31 and VE-Cadherin and similar levels of GLUT1 in hCMEC/D3-MDR1s versus hCMEC/D3s quantified in **(C)** Student's t test was performed; **** *p* < 0.0001. Experiments were performed on three distinct passages and in technical triplicate (*n* = 9); representative images are shown.
