# Supplementary figures and images for: Induction of P-glycoprotein overexpression in brain endothelial cells as a model to study blood-brain barrier efflux transport
Source: Front Drug Deliv. 2024 Jul 5;4:1433453. doi: 10.3389/fddev.2024.1433453 (PMC11981641; doi:10.3389/fddev.2024.1433453)

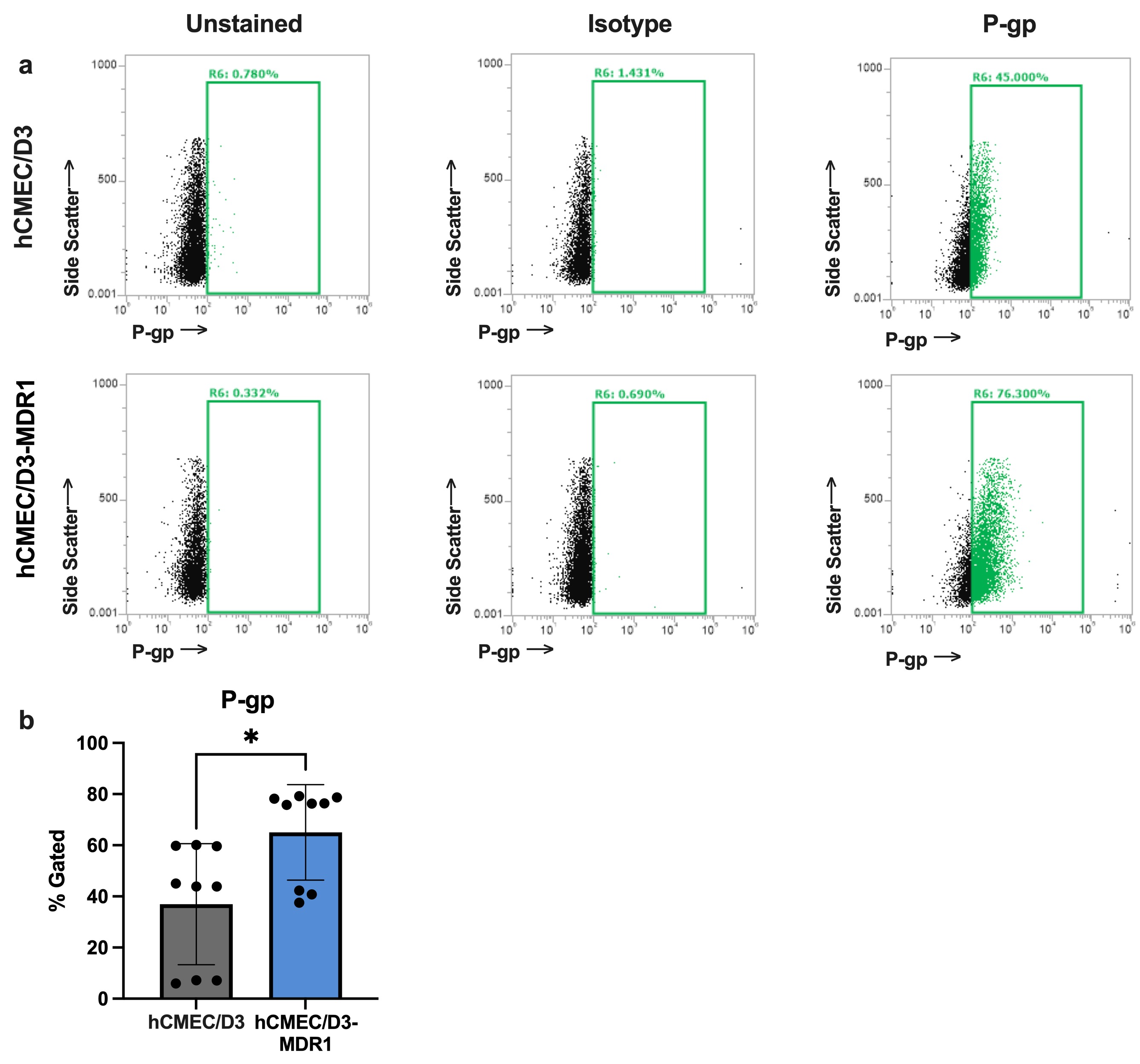

Supplement: Supplementary file 1 [file Image1.JPEG]

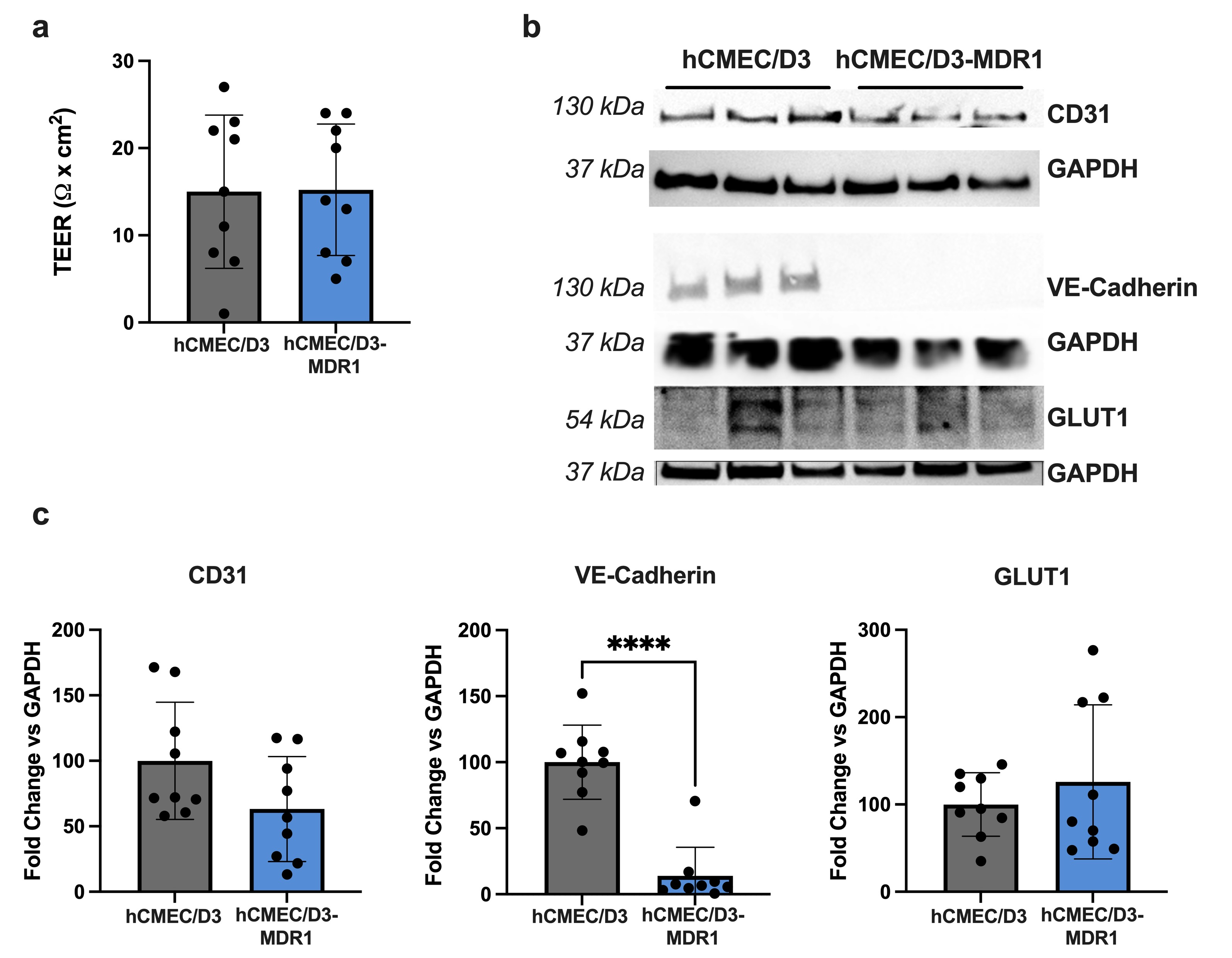

Supplement: Supplementary file 2 [file Image2.JPEG]
